# Nonerythroid Hemoglobin Present in Porcine Testes

**DOI:** 10.3390/ani15101352

**Published:** 2025-05-08

**Authors:** Jeffrey Li, Barbara Jean Nitta, Trish Berger

**Affiliations:** Department of Animal Science, University of California, Davis, CA 95616, USA; jlwli@ucdavis.edu (J.L.);

**Keywords:** hemoglobin, porcine, testis, juvenile, postpuberal, boar, interstitium

## Abstract

The presence of hemoglobin beta mRNA and protein in the female gonad suggests that hemoglobin beta may be present in the male gonad as well. The frequent occurrence of hemoglobin beta in nonerythroid tissues with hypoxic environments further underscores a potential role for hemoglobin beta in the testis to facilitate the regulation of oxygen availability for the developing germ cells and Sertoli cells since they are separated from the blood supply by multiple tissues. The presence of mRNA and protein were evaluated by qPCR and immunohistochemistry, respectively. The mRNA and protein for hemoglobin were detected in juvenile and postpuberal porcine testes. The most intense immunolabelling for the protein was present in testicular interstitial cells, in contrast to previously reported ovarian labelling in close proximity to the gamete and observed in porcine ovaries in the current study. The observed decrease in mRNA expression of hemoglobin beta with age is probably due to the change in testicular composition (increase in seminiferous tubule compartment) with age. The localization of hemoglobin beta in the testis will contribute to future understanding of its potential function in facilitating oxygen availability to seminiferous tubules or reducing oxidative damage.

## Simple Summary

Sperm development occurs in the seminiferous tubules within the testes. Sertoli cells and developing germ cells are dependent on oxygen diffusing from capillaries located within the interstitial tissue and diffusing through the interstitial tissue, followed by diffusion through or around the peritubular myoid cells and through the basement membrane. As a result of this long diffusion path, oxygen concentration within the seminiferous tubules is thought to be very low. The qPCR analysis demonstrated the presence of hemoglobin mRNA, consistent with previous reports of the presence of mRNA in the murine testis by microarray analysis. Immunohistochemical localization of protein in the interstitial compartment distinct from the vascular compartment confirms synthesis in this tissue. Although qPCR expression is lower in adult testis than in juvenile testis, the localization of protein in the interstitial compartment and the reduction in the proportion of interstitium with age may be the actual cause for the age-associated reduction in expression. The localization of the hemoglobin protein in the porcine oocyte and surrounding follicle confirmed a similar pattern to that recently reported for rodent and human follicles, supporting a significant difference in localization in gametes and proximal tissue between female and male gonads. This information on expression and localization provides a foundation for further experiments.

## 1. Introduction

Developing gametes are isolated from the immune system by blood gamete barriers (e.g., the blood–testis barrier that isolates secondary spermatocytes, spermatids, and spermatozoa from immune surveillance). Tight junctions between Sertoli cells protect the male gamete. Cumulus cells surrounding the oocyte provide a similar function in females. This isolation from the immune system means that oxygen diffuses from the blood vessels and through other cells before reaching the gamete itself, creating a relatively hypoxic environment [1]. The reduced temperature of the testes due to their scrotal location reduces the metabolic rate, which should ameliorate the severity of hypoxia [2,3,4,5]. However, oxygen availability may be near critical levels for developing spermatozoa. Observations that subfertile stallions had reduced testicular blood flow (hence reduced oxygen availability) compared with fertile stallions [6] suggested that oxygen availability is indeed near the critical level, supporting a role for nonerythroid (tissue) hemoglobin.

The presence of nonerythroid hemoglobin beta (HBB) is suggested to buffer tissue oxygen availability and/or guard against oxidative damage [7]. Recently, HBB appears to benefit oocyte development [8]. Parallels between the testis and ovary suggested to us that HBB might have an important role in testicular function as well. Hence, these studies were designed to evaluate presence and location of HBB in porcine testis. Potential involvement in testicular development was explored in juvenile boars with typical development and in littermates treated with letrozole, which inhibits aromatase, reducing estradiol and concurrently stimulating Sertoli cell proliferation. In addition, localization and relative expression were compared in postpuberal testes with that in juvenile testes.

## 2. Materials and Methods

### 2.1. Experimental Designs

The presence of the mRNA for *HBB* was evaluated by qPCR during testicular development. Developmental time points (1 week of age—neonatal, 5 weeks of age at the end of the first wave of Sertoli cell proliferation, and 20 weeks of age—a postpuberal time point characterized by sufficient motile sperm in the cauda epididymis for at least one insemination dose) were chosen. Animal treatment was previously described with animal protocols approved by the UC Davis Institutional Animal Care and Use Committee [9,10]. Testes from four neonatal boars, four 5-week-old boars treated weekly from 1 to 4 weeks of age with an oral dose of 1 to 2 mL of canola oil vehicle and three 20-week-old boars treated weekly from 1 to 6 weeks of age with an oral dose of 1 to 2 mL of canola oil vehicle were selected for qPCR analysis.

The presence of HBB protein was confirmed, and localization was evaluated using an immunohistochemical approach. Ovaries from two pubertal females were used as positive controls with labelling of the oocyte and cumulus cells as described in human cumulus oocyte complexes [11]. Testis tissues from seven young control animals (5–6.5 weeks of age), seven littermates to these controls treated with the aromatase inhibitor letrozole, and 10 postpuberal animals (20–40 weeks of age) were labelled with an antibody to HBB. Control animals were treated weekly with 1 to 2 mL of canola oil vehicle from 1 to 4 weeks of age for animals sampled at 5 weeks of age, treated from 1 to 5 weeks of age for animals sampled at 6.5 weeks of age, and treated from 1 to 6 weeks of age for animals sampled at 20 weeks of age. Littermates to the seven 5–6.5-week control animals were treated weekly with 0.1 mg letrozole/kg body weight in the canola oil vehicle from 1 to 4 or 1 to 5 weeks of age (for tissues recovered at 5 and 6.5 weeks of age, respectively). Letrozole treatment stimulates increased Sertoli cell proliferation at these time points [9].

### 2.2. qPCR

RNA was isolated from previously frozen testis tissue using the QIAzol Lysis Reagent^®^ (QIAGEN, Germantown, MD, USA). The RNA was DNase treated with RQ1 (Promega, Madison, WI, USA) before cDNA synthesis using a Revertaid Kit (Thermo Scientific, Waltham, MA, USA). The qPCR evaluation of *HBB* expression utilized *SPAG7* [12] as the reference gene, Lambda 2× qPCR Universal Green MasterMix (#qMX-Green, Lamda Biotech, St. Louis, MO, USA), and a QuantStudio 3 qPCR machine (Applied Biosystems, Waltham, MA, USA). The forward primer for *HBB* was CATTTGCTTCTGACACAACCGT, and the reverse primer was ACCACCAACTTCGTCCACATT. Efficiency for these primers was 93% (R^2^ = 0.92), with dilution and melt curves demonstrating a single product. The ΔCt was calculated from the mean of triplicates of *HBB* and *SPAG7*.

### 2.3. Immunohistochemistry

Testis tissues obtained from animals studied on animal protocols as previously described had been fixed in paraformaldehyde, dehydrated in a graded ethanol series, then vacuum infiltrated and embedded in paraffin. The 5 µm thick sections were transferred and dried on Superfrost Plus slides (Thermo Scientific, #22-034-979). Sections were dewaxed, rehydrated, and subjected to antigen unmasking, incubated with hydrogen peroxide in methanol to block endogenous peroxidase activity, further blocked with normal goat serum, and incubated overnight with a 1:200 dilution of the primary antibody (bs-8554R; Bioss, Woburn, MA, USA) in phosphate-buffered saline [13]. Negative control sections were incubated with a 1:10,000 dilution of normal rabbit serum in place of the primary antibody. The two rinses following incubation with the primary antibody (and after incubation with the second antibody and the first two rinses after the ABC reagent) contained 0.05% Tween-20 in the phosphate-buffered saline. Following incubation with biotinylated goat anti-rabbit secondary antibody (Vectastain^®^ Elite^®^ ABC Kit, #PK-6101; Vector Laboratories, Newark, CA, USA), the signal was amplified with ABC reagent and labelling was visualized with the AEC Peroxidase Substrate Kit (#SK-4200, Vector Laboratories).

### 2.4. Analysis of Labelling

Labelling intensity of interstitial tissue and seminiferous tubules was scored from 0 to 5 [14] by comparing each section with a reference scale created with sample images. Observer was blind to treatment. For each sample, labelling intensity scores for 10 sections were averaged; intra-assay CV was < 10% for mean scores from 10 sections.

### 2.5. Statistical Analysis

Data were subjected to analysis of variance (ANOVA) using R Statistical Programs [15], version 4.1.1 (aov function) with TukeyHSD used for posthoc pairwise comparisons. Data passed normality and homogeneity of variance tests. Age, location (interstitial vs. tubule), or treatment was a fixed effect. Data comparing response to letrozole treatment included litter in the model as animals were littermate pairs.

## 3. Results

**Figure 1:**
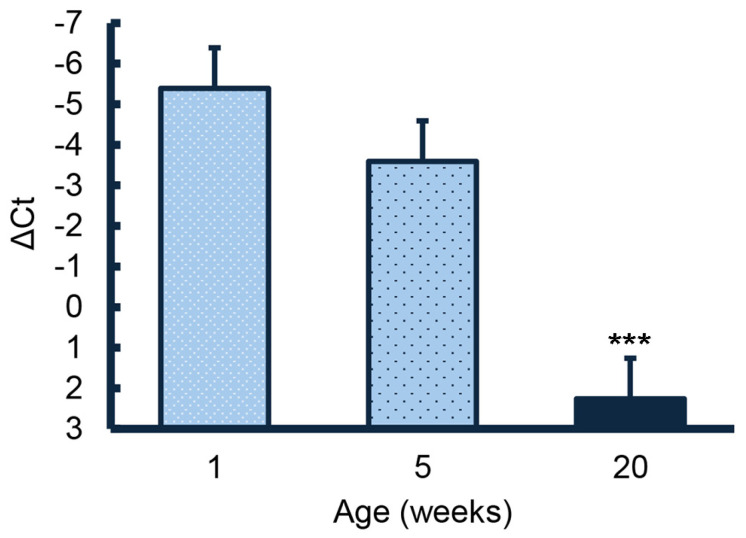
Expression of *HBB* mRNA in testes from 1-, 5-, and 20-week-old animals. Values represent means ± SE from 4, 4, and 3 boars with expression much lower at 20 weeks than in the juvenile animals. *** *p* < 0.001.

Expression of the HBB gene in the testis decreased with age. Expression in testes from the postpuberal 20-week-old animals was significantly lower (*p* < 0.001) than expression in testes from 1- and 5-week-old animals (Figure 1). Although expression in testes from 5-week-old animals appeared to be slightly less than expression in testes from1-week-old animals, values did not differ significantly.

**Figure 2:**
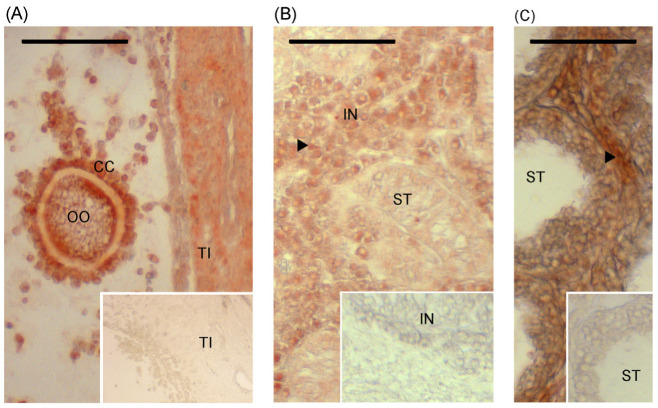
Immunolabelling of HBB protein in porcine ovary (**A**) and in testes from 6.5-week-old boar (**B**) and 24-week-old boar (**C**). The OO indicates a section of the oocyte located in the follicle with labelling detected in oocyte, cumulus cells (CCs), and lighter labelling in theca interna (TI). One of the testicular seminiferous tubules is indicated by ST in (**B**,**C**) and immunolabelling of interstitial tissue (IN) is indicated with an arrowhead. Insets represent the negative (normal serum) controls, and bars represent 100 μm.

**Figure 3:**
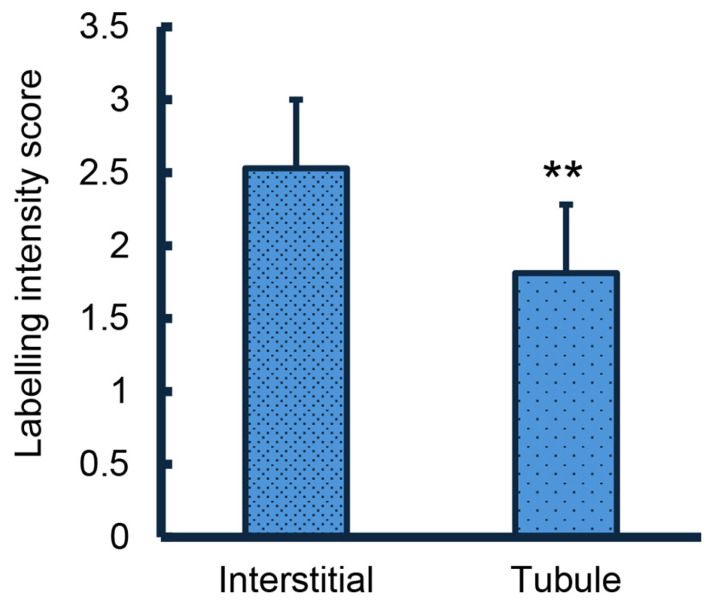
Immunolabelling for HBB protein demonstrated a stronger signal in the interstitial tissue than in the seminiferous tubules but faint immunolabelling was present in the seminiferous tubules. Values represent means ± SE of average labelling score of interstitial and tubule labelling in 7 juvenile and 10 postpuberal boars. ** *p* < 0.01.

Immunolabelling of HBB was readily detectable in the porcine ovary used as a positive control. The oocyte, cumulus, and granulosa cells were labelled, as indicated by comparison with the negative control (Figure 2A). Immunolabelling of HBB in young (Figure 2B) and postpuberal (Figure 2C) testes was observed with labelling clearly present in the interstitial space compared with the negative control. The intensity of labelling within the tubules was not affected by age nor was the intensity of labelling in the interstitial space affected by age, although the labelling intensity score was numerically higher in the younger animals on average (2.8 vs. 2.4, SE =0.6; *p* = 0.3). The intensity of labelling was higher in the interstitial space than within the seminiferous tubules (*p* < 0.01; Figure 3). This difference between interstitial tissue and the tubular compartment was also observed when juvenile and postpuberal boars were analyzed separately. Treatment with the aromatase inhibitor, letrozole, which prolongs the first wave of Sertoli cell proliferation beyond 5 weeks of age, did not affect the HBB labelling intensity of the interstitial space (2.8 vs. 2.6, SE = 0.5) or within the seminiferous tubules ((2.0 vs. 1.8, SE = 1.0).

## 4. Discussion

The decrease in the expression of HBB with age is consistent with the previous report of *HBB* following microarray analysis of murine testes [16]. The localization of the strongest signal for HBB protein in the interstitial compartment clarifies this apparent decrease in expression in the whole testis with age is due to the changing composition of the testis with age. The proportion of the parenchyma occupied by the tubular compartment increases as the seminiferous tubules increase in diameter and become filled with multiple generations of developing germ cells [17]. Consequently, the interstitial space occupies a smaller proportion of the testicular parenchyma. Genes expressed primarily in the interstitial space may have decreased expression with age simply due to the decreased proportion of interstitial space. We hypothesize that the low concentration of HBB present in the seminiferous tubules reflects diffusion of HBB into the seminiferous tubules and transfer with the developing germ cells into the lumens of the seminiferous tubules but cannot exclude local synthesis within the tubules. Although nonerythroid hemoglobins have been proposed to have a role in facilitating cell proliferation [18], no difference in HBB labelling was detected between testes from control animals and those from animals treated with letrozole to stimulate increased Sertoli cell proliferation [9]. Hence, these data do not provide evidence to support a role in Sertoli cell or germ cell proliferation.

The HBB labelling in the testis was more intense in the interstitial space than in the gamete compartment, in contrast to that reported for the murine and human ovary [11,19]. Labelling in the porcine ovary was similar to the human and mouse, being primarily associated with oocyte, cumulus and granulosa cells, confirming the gender difference in protein distribution. In the ovary, HBB has been suggested to facilitate oxygen availability in a hypoxic environment or to contribute to antioxidant capacity [20].

## 5. Conclusions

Increased synthetic activity within the seminiferous tubules (Sertoli cells or spermatozoa) did not appear to alter HBB protein expression. Although further research will be required to determine HBB function in the testes in facilitating oxygen or as an antioxidant, knowledge of its localization will contribute to this future understanding of its function in the male reproductive system.

## Data Availability

Data are presented within the text of this report and in the previously published, referenced papers.
